# Simple and Rapid Detection of Salivary Sheaths at *Philaenus spumarius* Feeding Points

**DOI:** 10.3390/insects17020229

**Published:** 2026-02-22

**Authors:** Aziza Husein, Valdete Sefa, Francesca Garganese, Ugo Picciotti, Giovanni Luigi Bruno, Maria Letizia Gargano, Francesco Porcelli

**Affiliations:** Department of Soil, Plant, and Food Sciences, University of Bari Aldo Moro, Via G. Amendola 165/A, 70126 Bari, Italy; aziza.hussein@uniba.it (A.H.); valdete.sefa@uniba.it (V.S.); giovanniluigi.bruno@uniba.it (G.L.B.); marialetizia.gargano@uniba.it (M.L.G.); francesco.porcelli@uniba.it (F.P.)

**Keywords:** OQDS, alien, invasive, quarantine organism, IPM, insect-borne plant pathogen, piercing–sucking mouthparts, plant–insect interactions, spittlebug, *Neophilaenus campestris*

## Abstract

Hemipteran plant sap feeders acquire food by inserting their piercing–sucking mouthparts into the apoplast (xylem sap feeders) or symplast (phloem sap feeders). When feeding, these insects seal their stylets inside the plant with a salivary sheath, minimising embolisms. The main impact of the insect on the host or food plant consists of sucking plant sap, transmitting phytopathogenic agents, or triggering galls as hypertrophic and/or hyperplastic neoformations. This paper proposes a rapid method for locating and counting the salivary sheaths of *Philaenus spumarius* (the Meadow Spittlebug), the primary Italian vector of *Xylella fastidiosa*, at the feeding point on its host or food plants. Hand-sliced stems of herbs hosting juveniles and olive twigs or leaves feeding the adult spittlebug, preserved while mounted in 75% ethanol (*v*/*v*), show the salivary sheaths. Alternative dyeing with acid fuchsin, chlorazol black, or phloroglucinol, and diaphanisation with Essig’s fluid alone or in combination with benzyl alcohol, may help with observations. The 75% EtOH solution provides a better compromise, yielding rapid evidence. The other methods offer similar outcomes but require more time and effort and expose the operator to harmful dyes. Assessing vectors’ access to plants may enable us to quantify the potential number of pathogen transmission events or to spot rejecting clones, thereby advancing the development of robust, effective control strategies that incorporate resistance.

## Simple Summary

*Philaenus spumarius* (*Ps*) is a xylem sap-feeding hemipteran that transmits *Xylella fastidiosa* (*Xf*) subspecies and a few other plant xylem-inhabiting microorganisms, including non-phytopathogenic ones. *Xylella fastidiosa* is a xylem-inhabiting olive pathogenic bacterium, the causal agent of Olive Quick Decline Syndrome in southern Italy. *Philaenus spumarius* secretes a proteinaceous saliva that jellifies into a salivary sheath to seal the stylets in place and maintain adequate fluid uptake. The salivary sheaths remain in situ within the plant tissues and reveal the hemipteran feeding point and path of a putative transmission event. Here, we compare several rapid and quantitative procedures for detecting *Ps* access and feeding via salivary sheaths. The number of accesses should be a key parameter for modelling the infection probability, given the estimates of the percentage of infective vectors in the study area. Moreover, given an orchard with diverse clones, counting salivary sheaths under the same vector population pressure will indicate comparatively fewer accepted plants, thereby supporting the search for resistant accessions.

## 1. Introduction

The Hemipteran Aphrophoridae are significant xylem sap-feeding vectors of microorganisms, including plant pathogens such as the quarantine bacterium *Xylella fastidiosa* Wells et al., 1986 (*Xf*), which threatens agriculture worldwide by inducing wilt and fatally dehydrating plants by blocking the water-conducting system [1]. The bacterium spreads only when adults access the xylem vessels to feed [2,3,4,5]. *Philaenus spumarius* (L., 1758) (*Ps*—Meadow Spittlebug) can also bear the *Candidatus* Phytoplasma fraxini [6], and *Ps* is also an alternative vector of *Candidatus* Phytoplasma solani [7].

*Xylella fastidiosa* entered Italy via asymptomatic ornamental coffee plants imported from Costa Rica [8], encountered potential vectors there, and initiated interactions with them. *Philaenus spumarius* adults currently play the main vector role, transmitting and spreading *X. fastidiosa* subspp. *pauca* ST53, which induces the epidemic OQDS in Apulian olive groves; *fastidiosa* ST1; and *multiplex* ST26 [9,10,11,12,13].

The meadow spittlebug is among the most polyphagous insects, feeding on approximately 1000 host and food plant species across 31 families [14,15]. The ability of *Ps* to enter diverse, relatively cold winter habitats makes it a key player in the transmission of xylem-borne plant pathogens, including *Xf* [6,16] in the Palaearctic. In warmer southern habitats, different Aphrophoridae, e.g., *Mesophthyelus* sp. [17], vicariate the vector *Ps*’s role.

*Philaenus spumarius* overwinters in egg batches, laid in recesses on herb residues [18,19]. The newborns appear in mid-February to March in Apulia [11,20]. The first naiads live on the basal leaf rosette, close to the leaf [21], stem, and soil surfaces [19,22]. Juveniles feed on available plants and, during post-embryonic development, crawl up the host plant as it grows [23,24]. Juveniles are not immotile and follow the host plant’s growth, feeding head down on the same or adjacent host herbs during post-embryonic development [25]. First juvenile instars secrete mucus and faeces while feeding, forming small bubbles in small ponds, not a true spittle [26]. First- and second-instar individuals aggregate in their own liquid mucus-and-faeces mixture. The third instar initiates whipping of the mixture to create a true spittle [26,27,28]. Many juveniles can share the same spittle near a herb knot, but they prefer to stay alone in a proper spittle while ageing, well protected in their own foamy mass from biotic and abiotic disturbances [11,14,18,29]. Many nymphs feeding on a small portion of the stem can cause wilting of the plant above the feeding points, rarely causing tissue necrosis [30].

Adult emergence occurs after about 7 weeks, from the herb tips, through the degrading spittle, which opens a chamber that protects the teneral adult [24]. In Apulian field conditions, the herbs dry at the beginning of olive flowering [31] in late spring and early summer [32], pushing *Ps* adults to tender olive twigs and leaves, where they can acquire and transmit *Xf* [11]. The drying of the meadows also forces *Ps* adults to circulate across the countryside and feed on actively vegetating shrubs and trees, abundant sources of xylem sap. By late summer or early autumn, the adults return to the newly regrown meadows, and females lay eggs for the next brood (oviposit) on plant remains [11].

*Philaenus spumarius* seals the stylets to the plant tissue and fills the gap among them with saliva, thereby maintaining hydraulic continuity and preventing embolism during sap uptake [33]. The saliva jellifies into persistent salivary sheaths that remain in place after feeding and stylet retraction, providing a time-persistent, detectable trace of the feeding events in the plants [20]. The sheaths demonstrate *Ps*’s adaptation to feeding on sap under high negative pressure (tension) that exceeds 200 times the *Ps* body weight in a day [34,35].

*Philaenus spumarius* is a multiplier of *Xf* infections, spreading the phytopathogen temporally and spatially [11]. When a non-infective *Ps* feeds on an *Xf*-infected olive plant, it can acquire the bacterium [36], becoming infective for life and transmitting *Xf* to other food plants. Detecting the salivary sheaths of xylem feeders is crucial for estimating the number of *Xf* transmissions, given that the percentage of *Ps* infective vectors in Europe ranges from 5 to 33% across surveyed specimens [37,38,39,40,41].

Wiegert [30] studied the feeding behaviour of *Ps* on tomato and used stains to spot the feeding points of *Ps* nymphs. Leopold et al. [42] stained the salivary sheaths of *Homalodisca coagulata* (Say, 1832) (Hemiptera: Cicadellidae) on *Chrysanthemums* sp., sunflowers, and *Hibiscus* sp. and found the ends of the sheaths in the xylem tissue and in the parenchyma. Cornara et al. [43] also investigated the feeding behaviour of *Ps* on olive trees using micro-CT to unveil the stylet track.

Our study focuses on developing and validating techniques to collect, preserve, and observe salivary sheaths at access on different *Ps* host and food plants. We aimed to refine the proposed methods and provide more detail on the salivary sheaths of *Ps* and other vectors. The ability of *Ps* to transmit *Xf* is beyond the scope of this study, but quantifying *Ps* feeding access will help in modelling the probability of pathogen transmission in the field. Studying vector access to plants may help identify undesirable or rejected clones and, in turn, highlight potential sources of resistance for incorporation into olive selection programmes. Finally, salivary sheath detection supports fast, precise, and quantitative infection management strategies to limit *Xf* outbreaks.

## 2. Materials and Methods

### 2.1. Survey Sites, Plant Material Collection, and Storage

We collected herbs in Valenzano (Bari, Italy) in the “P. Martucci” Agricultural Experimental Farm of the University of Bari Aldo Moro (Department of Soil, Plant and Food Sciences, DiSSPA), obtaining material in March–April 2024 and February–April 2025.

The plant identification follows Pignatti et al. [44,45,46,47], a standard floristic key for Italy, and nomenclature updated in accordance with POWO [48] and current taxonomic standards.

**Figure 1:**
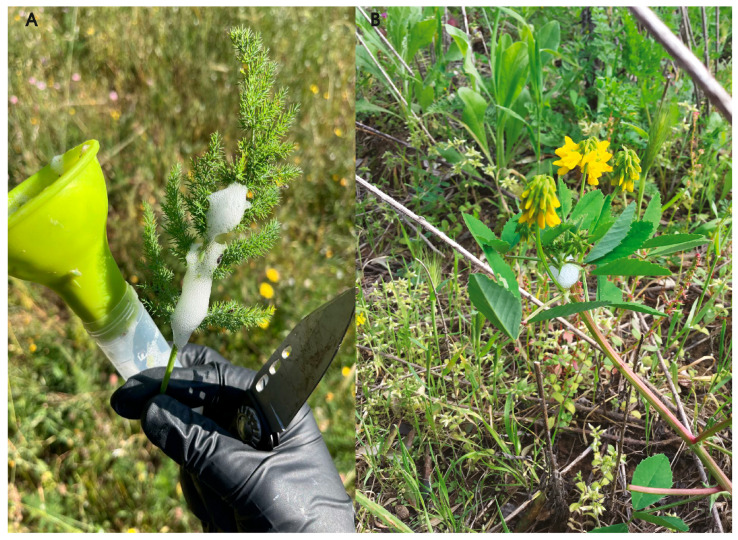
Collecting spontaneous herbs hosting *Philaenus spumarius* juveniles: (**A**) *Ferulago sylvatica*; (**B**) *Trigonella esculenta*.

We collected a total of 300 herb stems from each plant, representing 20 different botanical species (Table 1, Figure 1). We obtained 15 cm-long stem segments containing the *Ps* juvenile spittle (Figure 1). Each segment was promptly placed in zip-lock plastic bags, moved to the laboratory, and preserved in 75% EtOH (*v*/*v*) (Sprintchimica S.p.A., Sieci, Italy) until further analyses.

We also collected olive twigs with leaves in Parabita, Lecce (Apulia, Italy), during May, July, and December 2024, and January 2025. We collected 200 twigs (15–20 cm long) from 100 olive clones, 2 per plant. We preserved all the collected olive material in 75% (*v*/*v*) EtOH as prescribed by the rules to reduce the phytosanitary risk of *Xf* spreading.

### 2.2. Processing Plant Material

#### 2.2.1. Slice Preparation and Imaging

The collected plant material rested in the laboratory in zip-lock plastic bags immersed in 75% EtOH (*v*/*v*), with the ethanol changed three times during the first week to prevent the ethanol from becoming diluted by water extracted from the plant and to ensure proper fixation. All the collected samples were preserved in 75% EtOH for at least one month before slicing. Later, we moved the collection into clean Petri dishes, one clone at a time, for slicing and further processing. A Gillette half-razor blade mounted on a wooden handle was used to cut the plant material into about 1 mm thick segments, and we replaced the blade after each segment. We performed all slicing with the plant parts immersed in 75% EtOH, and we replaced the ethanol after each collection. It takes about one hour to slice 15 cm of a plant twig segment.

We examined all the slide-mounted plant slices using a Zeiss Tessovar photomicrographic zoom system (Carl Zeiss, Oberkochen, Germany) to search for *Ps* salivary sheaths. Once found, we transferred slices with salivary sheaths into separate numbered vials containing alcohol for further analysis.

Moreover, we performed slice-dyeing or clearing to make the *Ps* feeding sites more evident. We sectioned and treated three slices per protocol as follows:

1. Acid fuchsin.
2. Essig’s fluid with chlorazol black.
3. Essig’s fluid with benzyl alcohol.
4. Phloroglucinol.
5. Preserved only in 75% EtOH.

During counterstaining, we monitored the progress of the plant tissues daily for 2 weeks.

All processing and material storage were conducted at room temperature (25 ± 1 °C) in the laboratory.

#### 2.2.2. Acid Fuchsin

After softening the plant tissue in 20% KOH (Sigma-Aldrich, St. Louis, MO, USA), we rinsed the slices three times with distilled water (*w*/*v*) [49]. We moved the slices into a 5% acid fuchsin (Sigma-Aldrich, St. Louis, MO, USA) staining solution, prepared by dissolving the dye in distilled water and filtering the solution through filter paper. The herb and olive twig slices were stained for 1 hour, then rinsed in distilled water to remove excess dye before being cleared in 80% lactic acid (Greensistem s.a.s., Foggia, Italy) overnight at room temperature [49].

Some slices were overstained, and we processed the plant material as before, but at a lower acid fuchsin concentration (1 or 0.1%) and for a shorter (15 or 5′) treatment time.

#### 2.2.3. Essig’s Aphid Fluid and Chlorazol Black

The slices were stained in a mixture of Essig’s aphid fluid [50,51] and 0.7% chlorazol black (Sigma-Aldrich, St. Louis, MO, USA) at room temperature, monitoring the darkening over 3 weeks. We examined each slice every three days to detect the stained *Ps* salivary sheaths.

#### 2.2.4. Essig’s Aphid Fluid and Benzyl Alcohol

We stained the plant slices in a 1:1 (*v*/*v*) mixture of Essig’s aphid fluid and pure benzyl alcohol (Sigma-Aldrich, St. Louis, MO, USA) at room temperature for 2 weeks. We examined each slice every three days to detect stained *Ps* salivary sheaths.

#### 2.2.5. Phloroglucinol

We prepared an 8% (*w*/*w*) phloroglucinol solution (1,3,5-trihydroxybenzene) (Sigma-Aldrich, St. Louis, MO, USA) in pure ethanol. We stained the slices by applying five drops of 8% phloroglucinol for five seconds and then adding five drops of 37% hydrochloric acid (HCl) (VWR Chemicals LLC, Radnor, PA, USA) for five seconds. We rinsed the slices with pure ethanol to highlight the xylem vessels in red. We preserved the stained slices in 75% EtOH.

### 2.3. Imaging Slices and Salivary Sheaths

**Figure 2:**
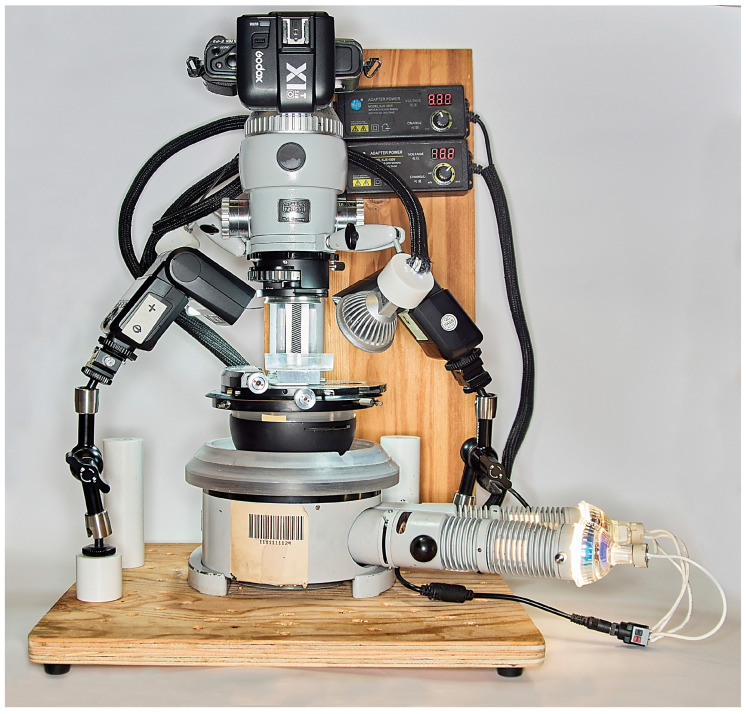
The Zeiss Tessovar photomicrographic Zoom System was used to image the plant sections.

We mounted microscopic slides, including the stained slices, in 75% ethanol. We observed and imaged the slides using the Zeiss Tessovar (Carl Zeiss, Oberkochen, Germany) (Figure 2), an Olympus PEN E digital camera (Olympus Corporation, Tokyo, Japan), a 2× Godox TT350S Mini Portable Speedlite, and a Godox X1R-N TTL 2.4G Wireless Flash Receiver (GODOX Photo Equipment Co., Ltd., Shenzhen, China). Two LED lights enabled proper focusing and framing via a USB connection to a digital monitor. We chose a Tessovar because it is a parfocal zoom lens optimised for close-up imaging, macrography, and micrography, with a magnification range of about 0.4×–12.8× (when used with the 4/3 sensor of the Olympus PEN E camera and the standard set of four matched auxiliary lenses). The obtained ORFs (Olympus Raw Files) were converted to JPG files using Capture One A/S (https://www.captureone.com/en, accessed on 3 March 2025).

After imaging the slices using light or electron microscopy, we returned them to 75% EtOH for long-term preservation and reuse in subsequent activities.

Selected slices in which salivary sheaths were observed by cryo-SEM using a TM3000 microscope (Hitachi, Tokyo, Japan), as in Picciotti et al. [52]. We cut the slices along the salivary sheath track, embedding them in ultrapure water (Milli-Q^®^ Lab Water, Sigma-Aldrich, St. Louis, MO, USA), which was frozen down to −45 °C at 10^−3^ Pa; ice sublimation and plant tissue unveiling were facilitated by rising the cryo stage T°C from −48 to −20 °C, and pictures were taken in “Analy” electron beam mode [53,54].

We processed the cryo-SEM images in Adobe Photoshop^®^ (Adobe, San Jose, CA, USA) using the Unsharp Mask filter with the following settings: Dust and Scratches radius = 2 pixels; Amount = 200%; and Gaussian blur = 0.3. We then adjusted the Levels (min = 0, max = 250) and isolated the background, cleaning it to achieve a uniform white. Finally, we applied false colours to represent the xylem, parenchyma, and salivary sheaths.

## 3. Results

### 3.1. Ethanol

**Figure 3:**
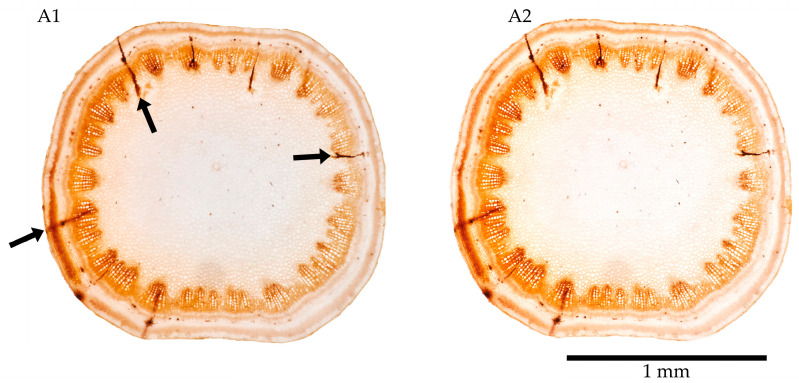
Stem slices of *Sonchus* sp. immersed in 75% EtOH, freshly prepared (**A1**) and still well-preserved after one month (**A2**). Black arrows mark salivary sheaths.

**Figure 4:**
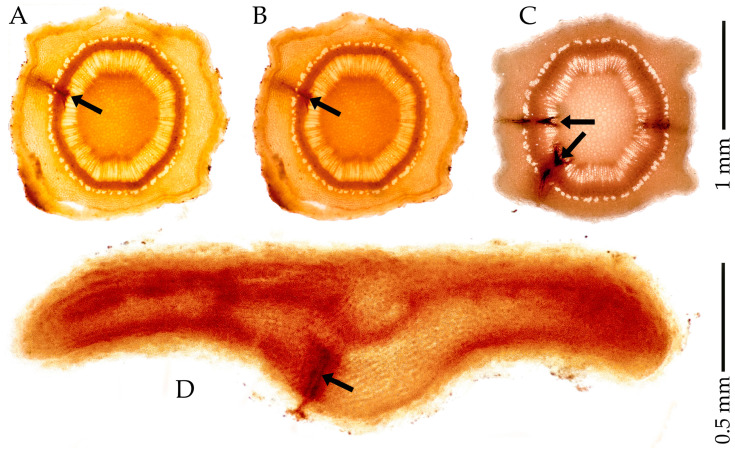
Slices from *Olea europaea* “Bella di Cerignola” CV twig (**A**,**B**); “Cima di Bitonto” CV twig (**C**); and “Sessana” CV olive leaf (**D**). All were preserved in 75% EtOH immediately upon slicing (**A**), after two months of preservation in 75 EtOH (**B**), or after one year of preservation in 75% EtOH (**C**). The “Sessana” leaf in (**D**) was preserved for one week. Black arrows mark salivary sheaths.

The salivary sheaths at the feeding sites in the spontaneous herbs and olive were visible in the collected stem and twig slices. They were promptly preserved in 75% EtOH (Figure 3(A1)) as dark dreads, contrasting with the surrounding clear plant structures. When preserved in 75% EtOH, these materials maintained their natural plant structure and salivary sheaths for one month (Figure 3(A2)) and remained well-preserved for up to one year (Figure 4C). A 75% EtOH solution worked for the basic preservation and detection of access points. The sections and the details they bear were generally stable and well-marked. Piercing stylets appeared radial to plant tissues, with the stylets tip branching to explore several neighbouring xylem vessels in the same xylem bundle. Attempts usually involve multiple intrusions into individual xylem vessels within a xylem bundle. However, incomplete feeding attempts occur, leaving shorter, tiny tracks in plant tissues that end in non-xylem vessels or bundles. Massive reactions to feeding are rare but do occur. Olive twigs and leaves were preserved in 75% EtOH for up to one year without any change in the plant tissues’ conformation or salivary sheaths’ appearance at the feeding sites (Figure 4), as expected. EtOH is the least hazardous preservative among those considered here.

### 3.2. Acid Fuchsin

**Figure 5:**
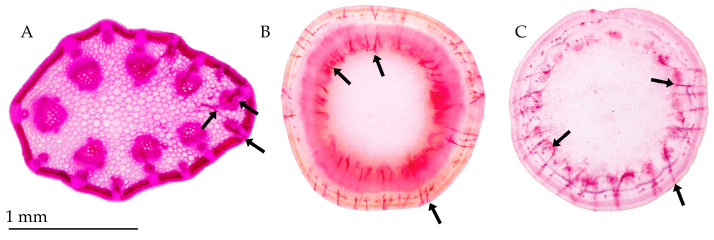
*Lotus* sp. slices of tip and larger stems stained with (**A**) 5% acid fuchsin, (**B**) 1% acid fuchsin, and (**C**) 0.1% acid fuchsin, all destained in 80% lactic acid, and stored in 75% EtOH. Black arrows mark salivary sheaths.

Salivary sheaths showed a dark pink to purple colour after 1 h in 5% acid fuchsin; acid fuchsin at 5%, 1%, and 0.1% successfully stained the salivary sheaths after 15 min, but also overstained some areas. A destain with 80% lactic acid improved the overall contrast and sheath evidence (Figure 5).

The staining procedures required one month of permanence in lactic acid without damaging the specimen.

Acid fuchsin stains fresh and 75% EtOH-preserved herbs or olive slices quickly and intensely, suggesting that concentrations as low as 0.1% can work. The correct concentration is unpredictable and depends on various plant and insect factors. Acid fuchsin often necessitates destaining in EtOH because it can overstain in actively growing host plant parts, and skin contact must be avoided.

### 3.3. Essig’s Aphid Fluid and Benzyl Alcohol

**Figure 6:**
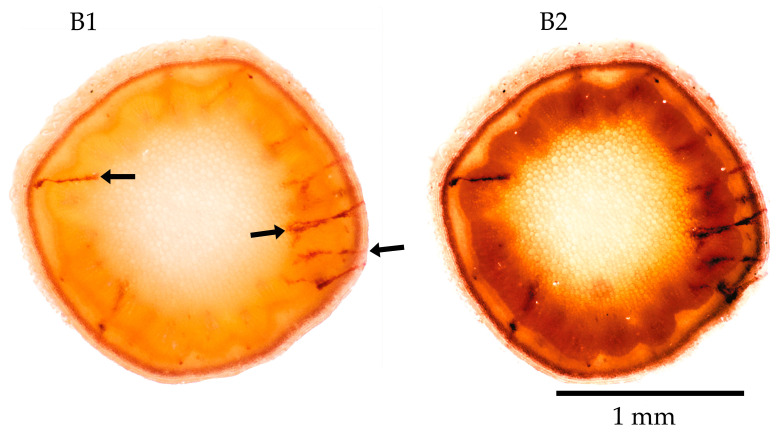
Stem slices of *Medicago* sp. immersed in Essig’s Fluid and benzyl alcohol (**B1**) and after 2 weeks in the mixture (**B2**). Black arrows mark salivary sheaths.

Essig’s aphid fluid [50] with benzyl alcohol (Figure 6(B1)) gives the entire slice an orange colour shift, which deepens to an intense orange after 2 weeks (Figure 6(B2)). In contrast, the *Ps* salivary sheaths assume a darker to black-orange shade. The Essig’s and benzyl alcohol mixture makes the plant tissue diaphanous and clarifies the plant’s anatomical details within a few weeks; see pictures in Figure 6(B1,B2). The operator must avoid skin or eye contact with the mixture because Essig’s fluid contains phenol, and the benzyl alcohol is also hazardous. Figure 6 clearly shows the *Medicago* sp. xylem bundles in the B1–B2 picture pair after two weeks of the same slice permanence in the mixture.

### 3.4. Essig’s Aphid Fluid and Chlorazol Black

**Figure 7:**
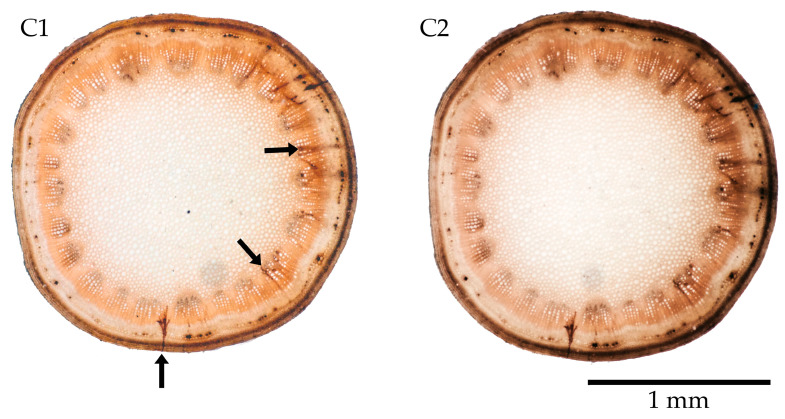
Stem slices of *Glebionis* sp. just immersed in Essig’s fluid and chlorazol black (**C1**), and after 1 week in the mixture (**C2**). Black arrows mark salivary sheaths.

Essig’s aphid fluid, amended with 0.7% chlorazol black, showed high specificity in staining salivary sheaths (Figure 7(C1)). After 1 week of storage in Essig’s aphid fluid and chlorazol black mixture, the salivary sheaths became darker, improving the contrast with the plant structures (Figure 7(C2)). However, chlorazol black is highly hazardous and may cause cancer, damage to unborns, and severe eye irritation. Also, it showed weaker differentiation between plant tissues and salivary sheaths among the tested samples.

### 3.5. Phloroglucinol

**Figure 8:**
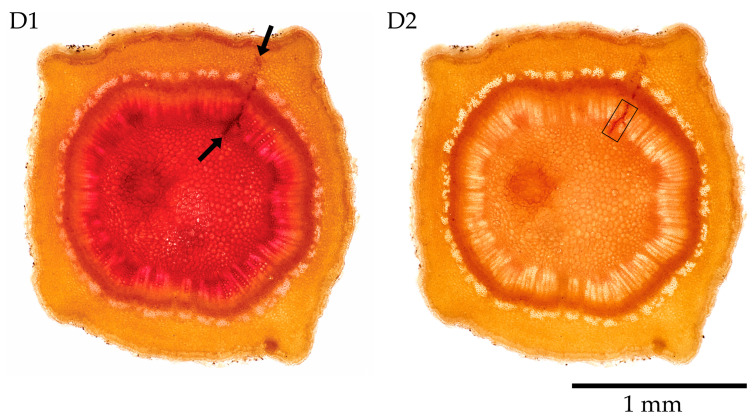
Transverse sections of *Olea europaea* L. F3P1 clone. Twig slices stained with phloroglucinol (**D1**), and 2 weeks later, after preservation in 75% EtOH (**D2**). Black arrows mark salivary sheaths; the frame points to the volume of plant tissue, partly shown in the cryo-SEM images, in Figure 9, Figure 10 and Figure 11 below.

The salivary sheath became reddish in slices stained with phloroglucinol (Figure 8(D1)). The slices preserved in 75% EtOH maintained the stain for a few hours (Figure 8(D2)), but they became more fragile and fainter over time. Phloroglucinol stains lignin red, wherever it is in the plant tissue. The salivary sheath’s colour faded relatively quickly, leaving the slice apparently untouched (Figure 8(D2)). The stain itself can cause skin irritation, allergic skin reactions, and serious eye and respiratory irritation. Moreover, the HCl used in the staining method can dissolve the cribro-vascular cambium, causing the tissue ring to detach from the cambium and attach to the bark/periderm. This issue occurs mainly on olive twigs.

### 3.6. Cryo-SEM of Twigs and Salivary Sheaths

To further demonstrate the presence of the salivary sheaths in situ and in agreement with the light microscopy evidence, we evidenced the volume of slices showing accesses in transverse sections, as shown in the inset of Figure 8(D2). We obtained clear evidence using the cryo-SEM technique [53,54], but it is difficult to appreciate in grayscale. To make the pictures clearer for readers, we rendered them in false colours.

**Figure 9:**
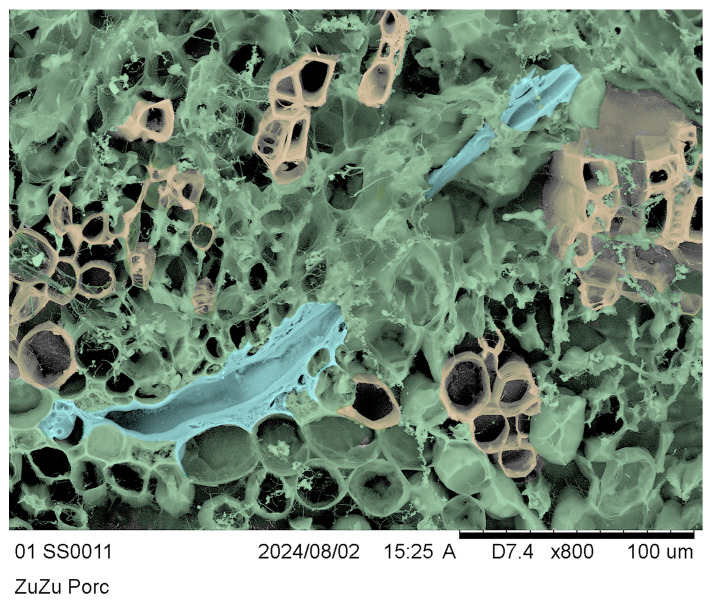
*Olea europaea* “Cima di Bitonto” CV transversal section false colours represent xylem in yellow, parenchyma in green, and salivary sheaths in pale blue.

**Figure 10:**
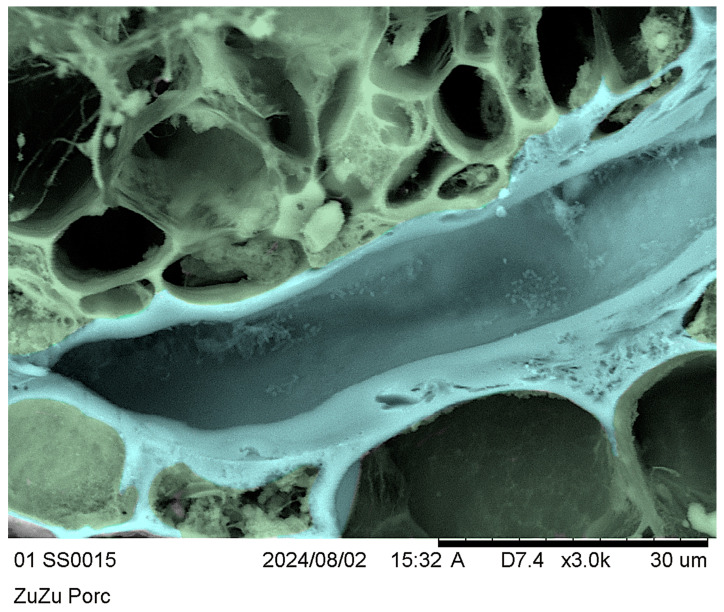
*Olea europaea* “Cima di Bitonto” CV transversal section: false colours represent parenchyma in green and salivary sheaths in pale blue.

Figure 9 shows a longitudinally cut salivary sheath segment (pale blue) intruding into the transversal slice section in the radial sense. We can also distinguish the xylem by morphology, differentiating the structural elements in yellow, parenchyma cells and contents in green, and the pale-blue salivary sheaths, which are sinuous and partially hidden by green parenchyma. Figure 9 and its detail in Figure 10 clearly show the amorphous matter of the salivary sheath, the vacant median duct resulting from the stylet’s retraction at the end of the feeding process, and granular matter contaminating the vacant median salivary sheath duct.

**Figure 11:**
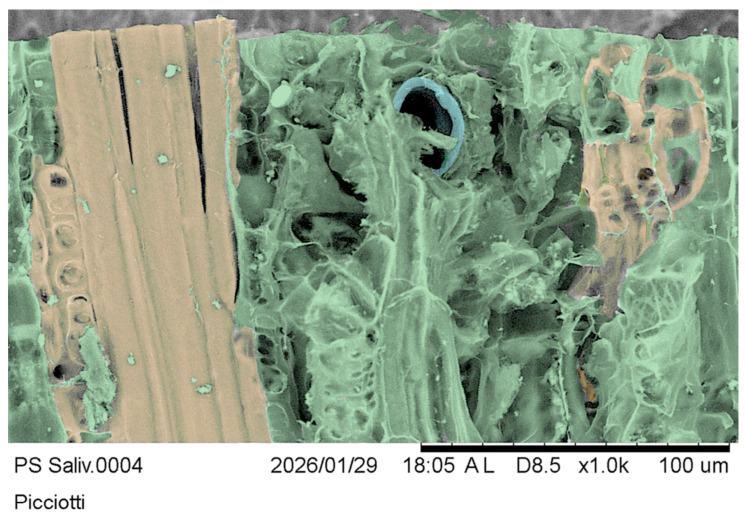
*Olea europaea* “Cima di Bitonto” CV tangential section: false colours represent xylem in yellow, parenchyma in green, and salivary sheaths in pale blue.

Figure 11 is a transverse salivary sheath section from a tangential olive slice section of the same inset matter in Figure 8(D2). The salivary sheath lining of the stylet-intruded duct appears somewhat elliptical. Still, the actual profile of the duct may differ due to artefacts, shrinkage, other stresses imposed on the plant morphology, and embedded insect secretion during preservation and preparation for observation.

## 4. Discussion

The *Xf* vectors transmit several plant pathogenic microorganisms by acquiring them from the xylem of infected plants and transferring them to uninfected host plants. The meadow spittlebug *Ps* is the primary vector of *Xf* in the Mediterranean [11,55]. Vector population censuses and analysing the dynamics play a primary role in developing and applying effective strategies to contain the pathogen’s outbreak. Integrated Transmission Management (ITM) manages timing and actions; it is essential for controlling *Xf* spread [11] in olive orchards through chemical [56], genetic [57], biological [55], agronomic [58,59], and physical means [60], targeting DSS-like tools for the prevention of *Xf* invasion.

Xylem sap feeders, including *Ps*, secrete their salivary sheaths during each feeding act on host or food plants. We propose various techniques for staining and detecting salivary sheaths in herbs and in olive twigs and leaves, both fresh and preserved. Our work highlights differences in specificity, clarity, and preservation over time among various techniques, while also improving previous methods for detecting salivary sheaths.

Methods exist to visualise the feeding tracks of xylem feeders. One of the first studies used safranin and fast green to confirm that *Ps* ingested xylem sap on tomato plant slices [30]. However, this method lacks the specificity of the safranin stain for different plant tissues, making it unsuitable. Meanwhile, the safranin did not provide sufficient contrast in the plant slice. Furthermore, the stain faded quickly, posing a challenge for finding alternative staining methods that offer better preservation and specificity.

Gerstenbrand et al. [49] investigated leafhopper feeding tracks on plants using acid fuchsin, demonstrating promising results for detecting feeding tracks but not for long-lasting ones. Acid fuchsin strongly stained feeding tracks in plant stems, but the stain faded over time. In contrast, our experience suggests that 75% EtOH is a suitable preservative, enabling subsequent simple, safe, and time-saving detections.

In comparison, Leopold et al. [42] used McBride’s stain to detect salivary sheaths in *Homalodisca vitripennis* (Germar, 1821) and lactic acid and phenol to clear the slices for observation. While McBride’s stain effectively highlighted the penetration sites of insect stylets, it showed limited contrast and faded over time. Our study used lactic acid to clear the plant twig slices. Still, it was time-consuming in comparison with using benzyl alcohol, which provided a more efficient clearing process. Cornara et al. [43] investigated the salivary sheaths of *Ps* on olive twigs and discovered one feeding access point. However, our applied techniques revealed multiple *Ps* accesses in both olives and herbs. Finally, Cornara et al. [61] assumed that the xylem anatomy of olives does not show a feeding preference, because examination of the Leccino and FS-17 varieties showed lower feeding susceptibility to *Ps*. Meanwhile, volatile organic compounds, visual cues, and organic acids, such as malic and isocitric acids, seem crucial for *Ps* host selection and could contribute to the tolerance of the olive cultivars Leccino and FS-17 (aka Favolosa) to *Xf* [61].

Overall, our results contributed to the optimisation of methods for observing the salivary sheaths of xylem-feeding insects. Acid fuchsin stained the feeding tracks. However, the 5% concentration overstained the slices, so we modified the stain concentration. Essig’s aphid fluid and chlorazol black specifically stained the insects’ salivary sheaths in the slices, but this process is time-consuming and requires a 2-week procedure. Staining with Essig’s aphid fluid and benzyl alcohol produced distinct colouration, with feeding tracks appearing in a darker orange shade than the other parts of the stained slice. However, using Essig’s aphid fluid and benzyl alcohol mixture is time-consuming and results in brittle slices. Phloroglucinol was effective in staining the xylem vessels and the salivary sheaths. EtOH preserved the staining, but distilled water and Essig’s aphid fluid affected the tissues, making them fragile; the stain faded over time and disappeared by the end of the first week. Therefore, we suggest 75% EtOH preservation of plant material, resulting in dark-brown sheaths following stylet penetration and feeding, avoiding hazardous chemicals, and saving time for further observation.

The macro–micrographic and cryo-SEM techniques we propose allow for targeting feeding events and quantitatively assessing interactions between *Xf* vectors and their host or food plants. Namely, the approach effortlessly reveals the salivary sheath resulting from feeding acts or attempts, as the vectors’ four stylets enter the plant feeding points. When we consider the techniques effective for counting the total number of feedings per twig and, thus, the worst-case first transmission per twig, we can estimate the possible number of transmissions per plant/year in each environment. The “worst case” [55,56] we tailor for infection will help with infection modelling, now suggesting that all xylem sap accessions belong to *Philaenus spumarius*. The assumption can be partial because several non-vector or candidate xylem sap feeders are present in our orchards. Still, we hope to identify the species and population sizes of xylem sap feeders using molecular techniques.

We can propose evaluating relative host/food plant preferences in candidates or vectors, by counting the number of accesses per plant per organ per year at the end of the growing season [31] in homogeneous orchards hosting clones/CV collections, to characterize the relative resistance/tolerance among available plants. Moreover, we should be able to quantify the relative food plant acceptance triggered by wood hardening at the end of the olive-growing season, using a dataset to better quantify the available transmission interval for *Xf*. A better understanding of the timing of plant susceptibility to xylem-sap feeders may help us focus on food plant clones/CV with earlier development, thereby decoupling food plant and vector phenology. The apparently simple and obvious techniques we propose may have several positive impacts on the quest for infection control through clonal selection, helping us to select food plants that discourage acquisition and infection.

## 5. Conclusions

Different methods helped us to scrutinise the feeding acts of *Xf* vectors in herbs, olive twigs, and leaves. All the procedures successfully stained the feeding accesses: acid fuchsin stained the entire plant slice, with darker staining at the feeding sites, but the stain was dark and difficult to remove at higher concentrations, necessitating clarification using lactic acid.

Using 75% EtOH enabled the detection of feeding tracks without staining. The mixture of Essig’s aphid fluid and chlorazol black specifically stained the insects’ feeding sites. In contrast, a mixture of Essig’s aphid fluid and benzyl alcohol changed the entire slice colour to darker tones over feeding tracks. Phloroglucinol highlighted red lignin parts, also masking accesses and eventually compromising the slice’s integrity.

The macrographic, micrographic, and cryo-SEM techniques we propose target feeding events and provide details of interactions between *Xf* vectors and their host or food plants, enabling easy, quantitative assessment. The “worst case” approach we tailor for infection will help in infection-event modelling, assuming that all xylem sap accesses are made by *Philaenus spumarius*. A better understanding of the timing of plant susceptibility to xylem sap feeders may help us to focus on food plant clones/CV with earlier development to uncouple food plant and vector phenology. The apparently simple and obvious techniques we propose will allow for the mass scrutiny of feeding events, which should have several highly positive impacts on the need for infection control by clonal selection, helping to target food plant selection that discourages acquisition and infection.

## Figures and Tables

**Table 1:** Herb species collected.

Family	Scientific Name
Rosaceae	*Rubus ulmifolius* Schott
Apiaceae	*Ferulago sylvatica* (Besser) Rchb.
Fabaceae	*Trigonella esculenta* Willd.
	*Lathyrus ochrus* (L.) DC.
	*Lotus* sp. L.
	*Medicago orbicularis* (L.) Bartal.
	*Trifolium medium* L. subsp. *medium*
	*Vicia lutea* L.
Asteraceae	*Sonchus oleraceus* L.
	*Sonchus asper* (L.) Hill
	*Calendula arvensis* L.
	*Galactites tomentosus* Moench
	*Andryala integrifolia* L.
	*Carduus pycnocephalus* L.
	*Glebionis coronaria* (L.) Cass. ex Spach
	*Glebionis segetum* (L.) Fourr. *Glebionis* sp. Cass.
Plantaginaceae	*Plantago afra* L.
Poaceae	*Phalaris canariensis* L.
Malvaceae	*Malva parviflora* L.

## Data Availability

Data is contained within the article.

## Abbreviations

The following abbreviations are used in this manuscript:

EtOH	Ethanol
IPM	Integrated Pest Management
ITM	Integrated Transmission Management
OQDS	Olive Quick Decline Syndrome
*Ps*	*Philaenus spumarius*
*Xf*	*Xylella fastidiosa*
KOH	Potassium Hydroxide
SEM	Scanning Electron Microscope
